# One in five patients require conversion to arthroplasty after non-vascularized bone grafts in patients with osteonecrosis of the femoral head: a systematic review

**DOI:** 10.1186/s13018-023-03544-8

**Published:** 2023-01-31

**Authors:** Jianxiong Li, Liang Mo, Guowen Bai, Zhangzheng Wang, Hua Zhang, Jie Li

**Affiliations:** 1 Guangzhou University of Chinese Medicine, Guangzhou, Guangdong China; 2 The First Affiliated Hospital of Guangzhou University of Chinese Medicine, Guangzhou, Guangdong China

**Keywords:** Meta-analysis, Osteonecrosis of the femoral head, Non-vascularized bone graft, Total hip arthroplasty, Hip preservation

## Abstract

**Background:**

Non-vascularized bone grafting (NVBG) has demonstrated to treat osteonecrosis of the femoral head (ONFH). There are a number of articles updating the use of NVBG to treat the ONFH, but the percentage of patients subsequently undergoing a total hip arthroplasty (THA) is controversial.

**Methods:**

Several electronic databases, including PubMed, Embase, Web of Science, and Cochrane databases, were searched to find studies using NVBG to treat ONFH. The pooled rate and 95% confidence interval (CI) were used to assess the conversion rate to THA after NVBG. In addition, we performed subgroup, sensitivity, and publication bias analysis.

**Results:**

A total of 37 studies describing 2599 hips were included. The mean weighted follow-up time was 50.5 months and the mean age at surgery was 36.3 years. The conversion rate to THA after NVBG was 21% (95%CI: 17% to 25%), and subgroup analyzes indicated lightbulb, trapdoor and Phemister techniques incidences with THA of 15%, 19%, and 24%, respectively.

**Conclusions:**

This study preliminarily obtained the general trend of the survival rate of NVBG patients, but these results should be interpreted cautiously. Pooled results from 2599 hips and of these nearly 80% with early stage of osteonecrosis, showed that approximately 21% of patients underwent a THA following NVBG. NVBG treatment for patient with ONFH appears to defer or at least delay the need for THA.

**Supplementary Information:**

The online version contains supplementary material available at 10.1186/s13018-023-03544-8.

## Introduction

Osteonecrosis of the femoral head (ONFH) is a refractory and high disabled hip disease, which primarily encounters young adults. ONFH most commonly arises from trauma, corticosteroid, alcohol use, blood dyscrasias and idiopathic necrosis of unknown causes [1]. The efficacy of different surgical treatments for ONFH and the influencing factors on prognosis are still under discussion [2–6]. Total hip arthroplasty (THA) is the treatment of choice for advanced-stage femoral head collapse [7]. Many surgeons typically prefer to delay performing THA, leaving THA as a last resort, because young patients undergoing THA usually need to accept hip revision or even multiple operations [8]. Importantly, the development of diagnosis technology has allowed the early diagnosis of ONFH, which provides more opportunities for hip preservation surgery. Therefore, increasing attention has been given to hip-preserving operations [9].

Since Phemister first used non-vascularized bone graft (NVBG) to treat ONFH [10]. Over the past decades, NVBG has demonstrated to be a viable treatment means for patients with ONFH, especially for pre-collapse (ARCO stages I and II) or early post collapse lesions (ARCO stage III) [11]. It can provide sufficient supporting structure after decompression of necrotic area and removal of necrotic bone, so as to promote the remodeling and healing of subchondral bone [12]. The hip survival rate was usually used to assess the effect of hip preservation surgery. It is recognized that despite most patients who undergoing upfront NVBG treatment subsequently need to go on to have arthroplasty, which may be considered a failure of the NVBG, NVBG may be considered successful by deferring the need for THA until later in life. Currently, multiple studies reported on NVBG for treatment of ONFH, but the clinical outcomes varied widely. Therefore, we aimed to make a quantitative analysis to assess the effect of NVBG in the prevention of THA in patients with ONFH.

## Materials and methods

### Search strategy and criteria

A comprehensive database search was performed by two reviewers (ML and LJ), including databases searched from PubMed, EMBASE, Web of Science, and Cochrane databases. Studies published from inception until May 1, 2022 were reviewed. The following search terms were used: “femur head necrosis” or “avascular necrosis of femur head” or “ischemic necrosis of femoral head” or “aseptic necrosis of femur head”, and “bone transplantation” or “bone grafting” or “transplantation bone” or “allografts”. Besides, a manual review of references from eligible systematic and other review articles was performed to ensure no eligible studies were omitted.

Full-text articles were selected according to the following inclusion criteria: (1) Human studies in English language from inception until April 25, 2022; (2) Minimum level IV case series studies using Oxford Center for Evidence-Based Medicine 2011 Levels of Evidence; (3) Established diagnosis of ONFH, outcomes together with NVBG technique were reported; (4) At least 10 hips were evaluated. The articles were excluded according to the following criteria: (1) Non-English articles; (2) Any type of augmentation was used (e.g. vascularized bone grafts or bone marrow stem cells); (3) The mean follow-up time less than 24 months; (4) Review/purely technique articles/animal studies.

### Data extraction

Two reviewers independently extracted the following information from the included studies: the first author; publication year; the number of patients and hips; sex ratio; level of evidence, surgery technique; stage (ARCO or Ficat or Steinberg); radiological outcome; clinical outcome and follow-up time.

### Quality assessment

Level-of-evidence rating was extracted for the included studies based on the “Oxford Center for Evidenced-Based Medicine—levels of evidence”. In addition, the Newcastle–Ottawa Scale (NOS) was used to assess the methodologic quality of the included case–control and cohort studies [13].

### Sensitivity and statistical analysis

Where appropriate, a sensitivity analysis was performed by excluding one study at a time to weight up the relative influence of each individual study on the pooled effect size. Statistical analyses were performed using metaprop packages in Stata statistical software version 14.0 [14]. In order to explore heterogeneity and evaluate studies based on possible confounders, forest plots were developed for calculation of effect size and confidence intervals (95%). For proportions of hips undergoing hip replacement, the datasets were developed from calculated individual proportions of studies and their confidence intervals. Heterogeneity among studies was assessed by I^2^ using the standard Chi-squared test. Values less than 50% represent mild to moderate heterogeneity and a fixed effects model was used, whereas values greater than 50% represent substantial to considerable heterogeneity and a random-effects model was used. The funnel plot was used to assess publication bias, which was identified by an asymmetry in the funnel plot.

## Results

### Search results

**Fig. 1:**
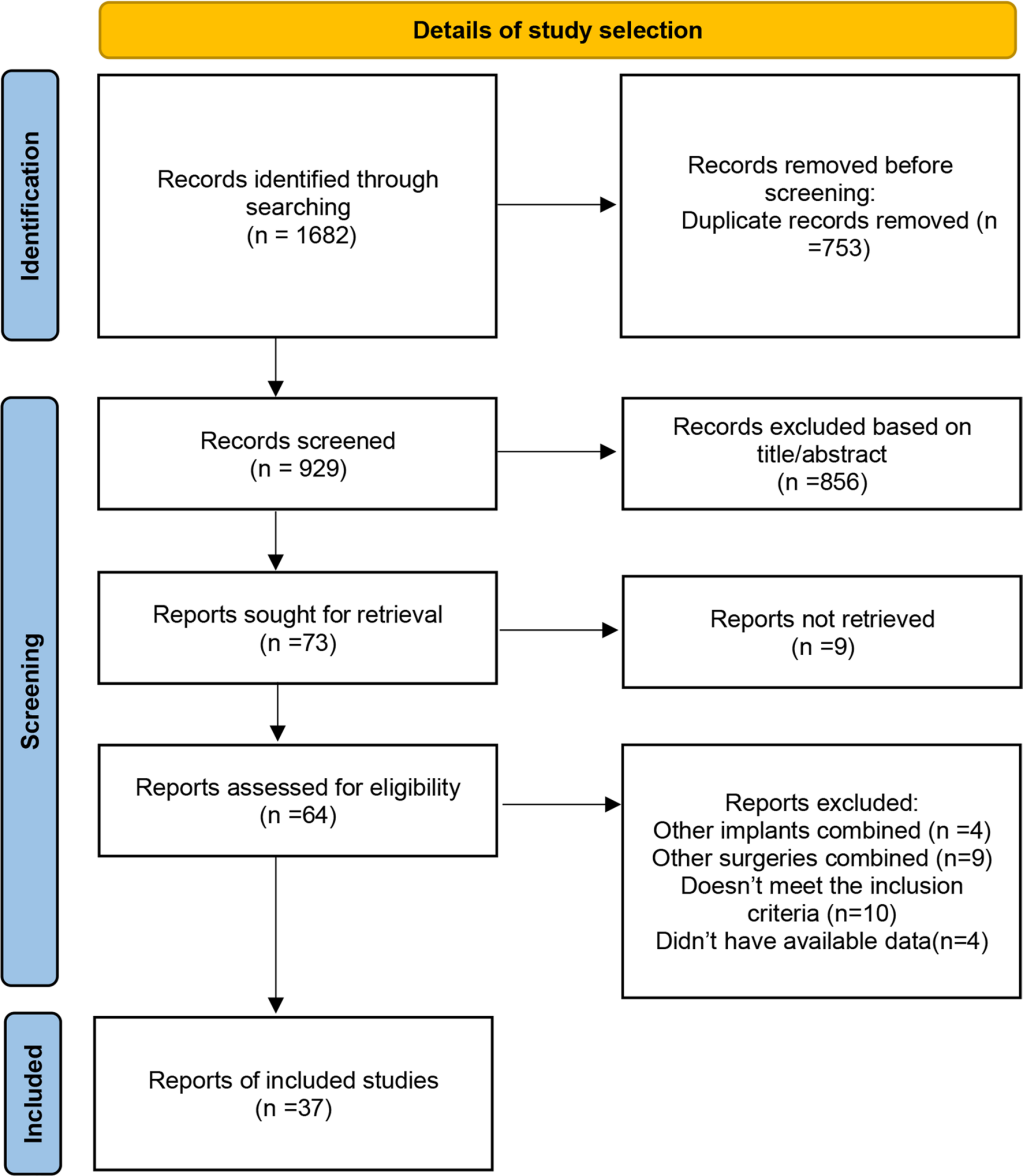
Flowchart of included studies

A total of 1682 studies were identified by the preliminary literature search, of which 753 duplicate articles were excluded. Finally, 37 articles were included after layer-by-layer screening. No extra articles were eligible for inclusion from the references lists in the retained articles. The search and exclusion process are shown in Fig. 1.

### Characteristics of included studies

Among the 37 selected studies, nine studies were case–control studies and 28 studies were cohort studies (Table 1). In addition, it’s interesting to find that studies conducted extensively in China, accounting for more than 50% (21/37) of the included studies, which may hint that China has a vast number of patients with ONFH and the number of patients far exceeds other countries. This has spurred the development of hip preservation surgery, including non-vascularized bone grafting.

**Table 1:** Detailed information of the includes studies

Study	Country	Patients	Hips	M/F	Age	Stage	Etiologies (patients or hips)	Bone graft materials	Technology	Follow-up(m)	Hips converted
Zhou [15]	China	64	70	51/13	32.0	ARCO II:8/III:62	SI:33/AI:19/IP:10/TM:8	Autogenous ilium	Lightbulb	60	9
Yue [16]	China	96	139	79/17	37.5	ARCO II:63/III:76	SI:66/AI:44/IP:20/TM:9	Allograft bone + hydroxyapatite bone	Phemister	29.26	18
Wu [17]	China	50	50	42/8	40.0	ARCO II:28/III:22	SI:8/AI:36/IP:6	Impaction bone with a wire coil	Lightbulb	109.2	19
Zhang [18]	China	16	17	42/8	31.5	ARCO II:14/III:3	NA	Autologous ilium	Phemister	29.27	4
Kuroda [19]	Japan	20	20	12/8	41.0	ARCO II:1/III:19	SI:12/AI:5/IP:1/SI + AI:2	Hydroxyapatite + autologous bone	Phemister	36.5	7
Liang [20]	China	47	47	39/8	38.3	ARCO II:24/III:23	SI:13/AI:21/IP:13	Autologous illium + bioceramic bone	Lightbulb	44.6	12
Jie [21]	China	34	50	30/4	41.6	ARCO II 32/III:18	SI:12/AI:19/IP:1/TM:2	Autologous fibular	Phemister	154.8	6
	China	83	103	66/17	35.7	ARCO II:67/III:36	SI:31/AI:29/IP:7/TM:16	Allogeneic fibular	Phemister	111.6	13
Chen [22]	China	54	64	44/10	35.4	ARCO II:14/III:50	SI:23/AI:28/IP:4/TM:5/SI + AI:4	Allogeneic fibular	Phemister	103	7
Yuan [23]	China	34	48	29/5	37.0	ARCO II:25/III:23	NA	Allogeneic fibular	Phemister	80.4	14
Chen [24]	China	44	44	29/15	36.5	Ficat II:44	SI:15/AI:26/IP:3	Allogeneic fibular	Phemister	88.8	8
Moon [25]	Korea	52	72	40/12	37.2	Steinberg I + II:49/III:23	SI:24/AI:38/IP:10	Multiple matchstick-like bone	Phemister	40.8	19
Cheng [26]	China	67	67	41/26	36.3	ARCO II:45/III:22	SI:20/AI:37/IP:10	Autologous ilium	Trapdoor	91.2	3
	China	72	72	43/29	38.5	ARCO II:46/III:26	SI:22/AI:38/IP:12	Autologous ilium	Lightbulb	72	9
Feng [27]	China	46	51	26/20	32.8	ARCO III:51	SI:20/AI:15/IP:11	Autologous ilium	Trapdoor	78	11
Wang [28]	China	66	66	52/14	38.1	ARCO II:66	SI:23/AI:34; IP:9	Autologous ilium	Lightbulb	48	10
Wu [29]	China	24	29	22/7	38.9	ARCO II:26/III:3	SI:11/AI:11/IP:7	Allogeneic fibular	Phemister	168	10
Lin [30]	China	16	16	12/4	32.6	ARCO II:8/III:8	TM:5/non-TM:11	Autologous illium	Phemister	36	3
Sionek [31]	Poland	53	58	45/8	35.5	Ficat II:38/III:20	SI:26/AI:32	Calcium phosphate bone substitute	Phemister	50.4	11
Stefan [32]	Germany	24	29	20/4	42.9	ARCO II:29	SI:15/AI:3/IP:6	Calcium phosphates + autologous bone	Phemister	30	7
Yildiz [33]	Turkey	21	28	14/7	33.2	Steinberg I:49/ II:12/III:23/IV:2	SI:15/IP:13	Autologous ilium	Lightbulb	52.6	4
Zuo [34]	China	119	158	86/33	33.2	ARCO II:27/III:131	SI:85/AI:21/IP:52	Autologous ilium	Lightbulb	31.1	31
Vahid [35]	Iran	96	132	57/39	33.7	ARCO II:121/III:66	SI:15/AI:19/IP:5/TM:48/Drug:12	Autologous ilium	Lightbulb	48.5	10
Yang [36]	China	35	46	23/12	37.1	Steinberg I:8/ II:32/III:6	SI:19/AI:5/IP:11	Autologous cancellous bone	Phemister	24	19
Gagala [37]	Poland	13	14	13/0	37.7	ARCO II:3/III:2/IV:9	SI:3/AI:5/IP:5	Autologous + allografts bone	Trapdoor	32.7	5
Zhang [38]	China	65	82	43/22	31.4	ARCO I:5/ II:66/III:14	SI:24/AI:28/IP:4/TM:9	Artificial bone + autogenous bone	Lightbulb	24	6
Wang [39]	China	25	28	23/2	39.9	ARCO I:2/ II:17/III:9	SI:2/AI:16/IP:7	Allogeneic fibular	Phemister	104.5	18
Wei [40]	China	162	223	101/61	33.5	ARCO II:134/III:89	SI:110/AI:90/IP:23	Allogeneic fibular	Phemister	24	43
Hsu [41]	America	31	62	20/11	40.6	Steinberg I–II	SI:15/AI:4/IP:10/Other:2	Graft demineralized bone	Phemister	46	23
Wang [42]	China	110	138	69/41	32.4	ARCO II:68/III:71	SI:99/AI:27/IP:12	Autologous ilium	Lightbulb	25.4	13
Chang [43]	China	11	11	9/2	37.0	ARCO II:5/III:6	SI:3/AI:7/IP:1	Autologous cancellous bone	Lightbulb	61	3
Keizer [44]	Netherland	80	80	56/24	36.0	Ficat I:9/II:48/III:13/IV:10	SI:48/AI:7/TM:15/Other:10	Allogeneic fibular and tibial grafting	Phemister	48	34
Kim [45]	Korea	19	23	15/4	44	Steinberg II:10/III:2/IV:11	SI:7/AI:9/IP:7	Autologous fibular	Phemister	48	5
Rijnen [46]	Netherland	27	28	21/6	33	ARCO II:11/III:14/IV:3	SI:10/AI:4/IP:7/TM:5/Other: 2	Autogenous bone and artificial bone	Phemister	24	8
Steinberg [47]	America	227	312	134/93	37	Steinberg I:69/II:133/III:13/IV:92/V:5	SI:107/AI:43/IP:32 SI + AI:34/Mixed:32	Autologous cancellous bone	Phemister	24	113
Mont [48]	America	22	30	16/7	26	Ficat III:24/IV:6	SI:16/AI:8/IP:3/Other: 3	Autologous cancellous bone	Trapdoor	56	8
Nelson [49]	America	40	52	32/8	NA	Marcus II:17/III:11/IV:22/V:2	SI:16/AI:18/IP:5/SI + AI:1	Autologous tibial grafting	Phemister	24	4
Buckley [50]	America	19	20	13/6	NA	Marcus I:1/II:19	SI:9/AI:2/IP:8	Autogenous and allogeneic bone	Phemister	96	2
Bakx [51]	Netherland	16	20	10/6	NA	Ficat II:4/II–III:5/III:9/IV:2	NA	Autogenous tibial bone grafting	Phemister	33	2

*M* male; *F* female; *SI* steroid-induced; *AI* alcohol-induced; *IP* idiopathic; *TM* traumatic

A total of 2100 patients were included in the study. There were 1519 males (72.3%) and 581 females (27.7%). The mean weighted follow-up time was 50.5 months and the mean age at surgery of patients was 36.3 years. The main etiologies of ONFH included the following: usage of corticosteroids and alcohol abuse (accounting for more than 65%), traumatic and idiopathic (accounting for nearly 30%). The techniques used for NVBG were mainly included Phemister (60.9%), lightbulb (32.9% of patients) and trapdoor (6.2% of patients). The bone graft materials commonly used in the surgical treatment of ONFH include autologous ilium, particulate bone graft, and allogeneic bone graft.

### Quality of the included studies

The methodologic quality of the included studies was assessed using the NOS and Level-of-evidence rating (Additional file 3: Tables S1 and S2). The methodologic quality of the included studies was relatively stable.

### Results of the meta-analysis

**Fig. 2:**
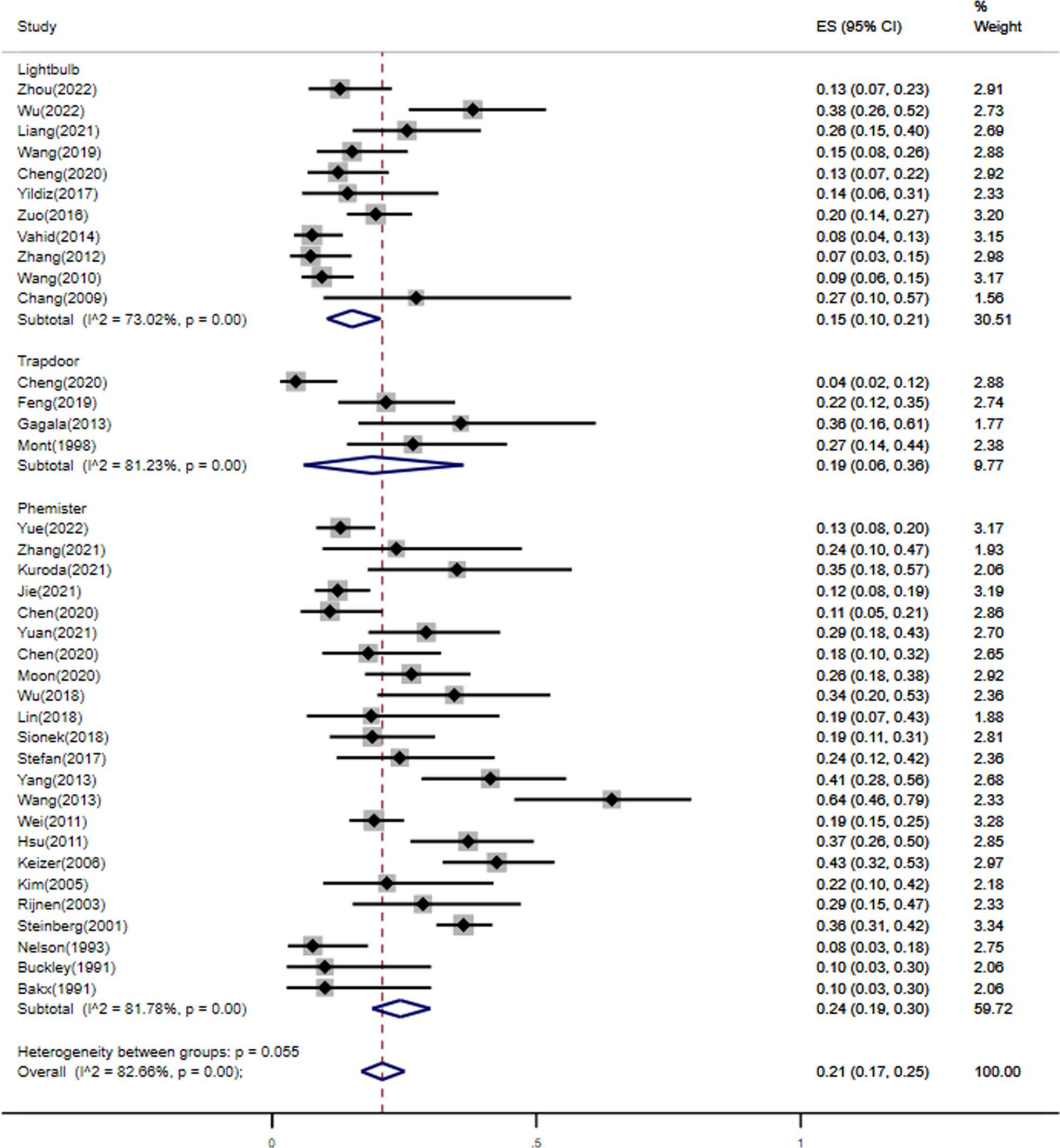
Proportion Forest plot of studies reporting percentage of hips undergoing THA after NVBG, by surgery techniques as analyzed by metaprop

37 studies reported that number of THA after NVBG and a total of 2100 patients (2599 hips) were included in this study, including 551 hips converting to THA after NVBG with the mean following time ranging 24 months to 154 months. The overall pooled proportion of hips undergoing THA was 21% (95%CI: 17% to 25%). As the heterogeneity test showed heterogeneity among the studies (*I*^2^ = 82.39%, *p* < 0.01), the random-effects model was used for the meta-analysis (Fig. 2).

When examining outcomes, it is important to consider the role of surgical technique. For further stratification, studies were separately evaluated based on the surgery technique: the pooled conversion rate to THA after NVBG was 15% (95%CI: 10% to 21%) for lightbulb technique, 19% (95%CI: 6% to 36%) for trapdoor technique, and 24% (95%CI: 19% to 30%) for Phemister technique (Fig. 2). But the heterogeneity was not demonstrably decreased. Therefore, we also conducted a subgroup analysis of follow-up time (≥ 5 years and < 5 years) (Additional file 1 Fig. S1). This could not, however, significantly reduce the heterogeneity, as all I^2^ values were above 60%, which may represent substantial heterogeneity regardless of the abovementioned stratification efforts.

**Fig. 3:**
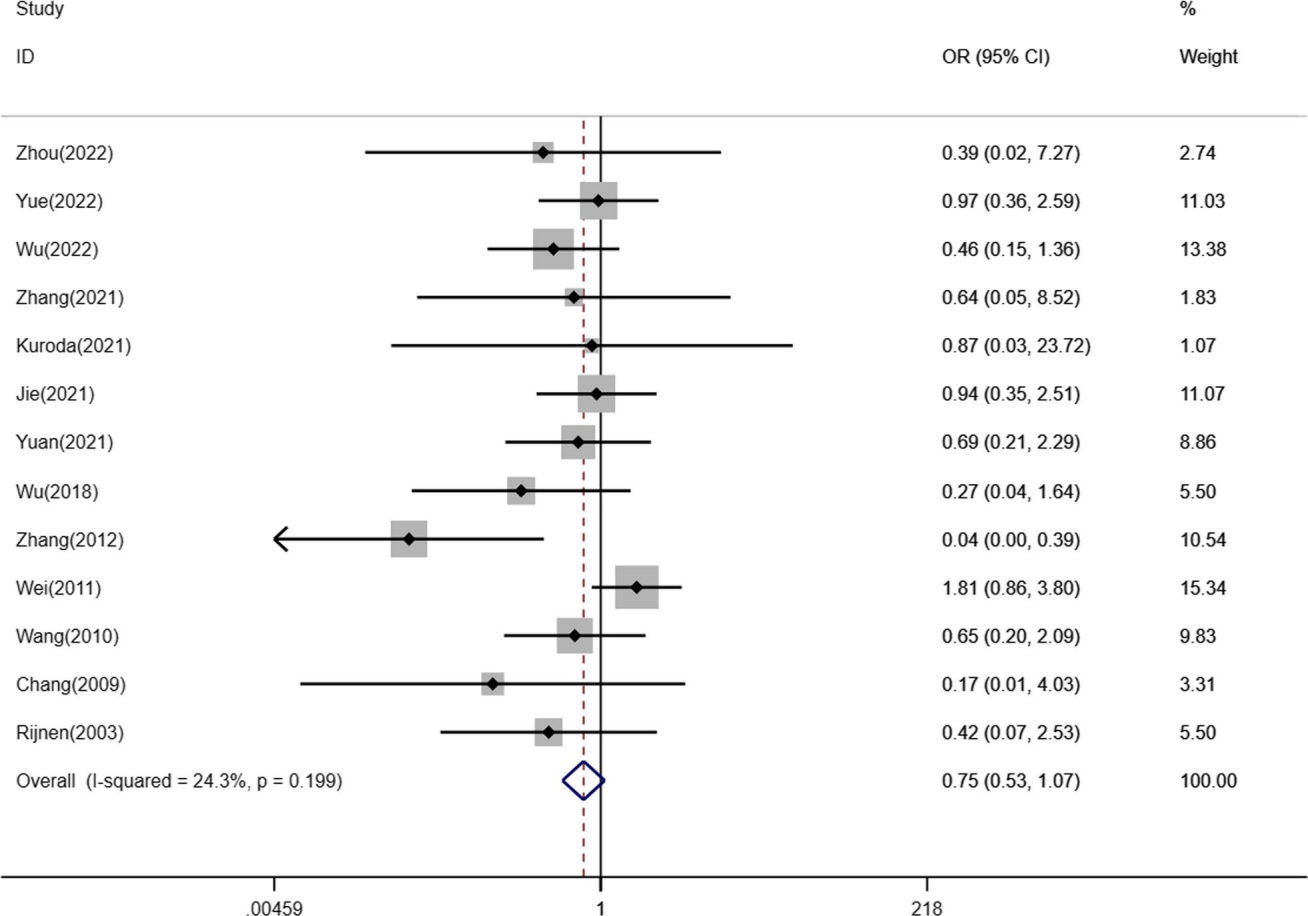
Forest plot for comparison of the conversion rate to THA between the ARCO II and ARCO III groups

**Fig. 4:**
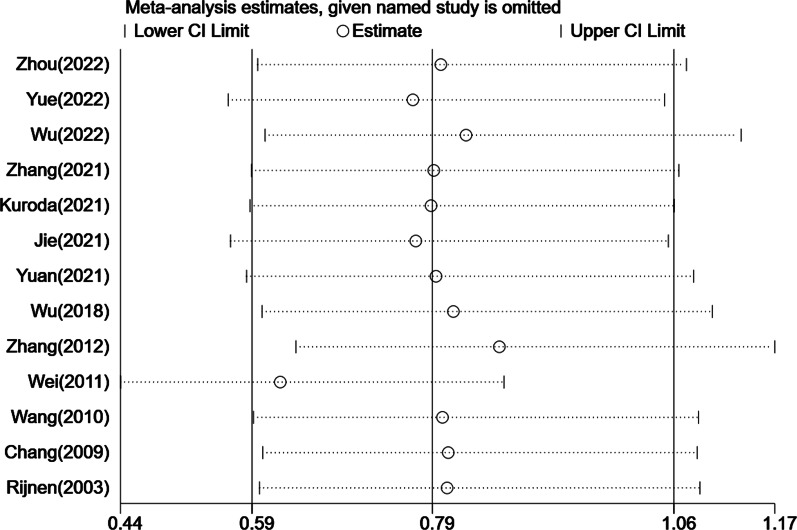
Influence analysis of included studies

In addition, of the 37 included studies, 13 studies reported the correlation between ARCO classification and THA after NVBG. The fixed effect model was chosen due to nonsignificant heterogeneity in intra-study comparisons (*I*^2^ = 24.3%, *p* = 0.199). No statistically significant difference in this index was shown between the ARCO II and ARCO III groups (OR: 0.75, 95%CI: 0.53–1.07, *p* = 0.112) (Fig. 3). At the same time, the sensitivity analysis was performed on the selected studies to assess whether individual studies would affect the overall results. The outcomes suggested that no individual study strongly affected the overall results (Fig. 4).

### .

#### Sensitivity analysis and publication bias

Sensitivity analysis was carried out on the 37 articles included in this meta-analysis, and no individual study caused significant interference with the results, indicating that this meta-analysis was stable. The shape of the funnel plot and Egger’s test (*p* = 0.03 < 0.05) revealed possible publication bias (Additional file 2: Fig. S2).

## Discussion

This study systematically collected clinical trials of patients with ONFH undergoing NVBG and conducted a meta-analysis and systematic review, which based on 2100 individuals and 2599 hips. The results revealed that the incidence of THA after NVBG in patients with ONFH was 21% the mean weighted 50.5 months follow-up time. In terms of surgery techniques, patients undergoing NVBG with lightbulb technique are at lower risk of conversion to THA (15%) than trapdoor (19%) and phemister (24%) techniques at the mean weighted follow-up time of 45.7 months, 75.5 months and 50.7 months, respectively. There was no significant difference in the incidence of THA between the ARCO II and ARCO III groups. Recently, Andronic et al. [52] performed a meta-analysis of core decompression alone in nontraumatic ONFH treatment, which showed that 38% of patients underwent a total hip replacement at an average of 26 months follow-up. Therefore, the hip survival rate of patients with ONFH after NVBG was acceptable in the middle term compared with the conversion rate of core decompression alone.

To our knowledge, there were no previous studies conducted to assess the conversion rate to THA after NVBG using quantitative meta-analysis. Our study represents an effort to summarize all the available evidence, which describes NVBG as a treatment for ONFH. Our study reveals that NVBG treatment for patient with ONFH appears to defer or at least delay the need for THA, and the risk of conversion to a THA is not very high in the middle term. However, this review could not determine whether NVBG can arrest disease progression due to lack of stratification and heterogeneity of data.

When bone grafts mentioned, the role of surgical techniques were also mentioned. The Phemister technique was the first NVBG technique described in 1949 [10]. The basic concept of the technique involves removing a 7- to 9-mm-diameter cylindrical core from the femoral head and neck, which is then replaced by a bone graft removed from the tibia, fibula, or ilium. In our study, the Phemister technique has been used in 23(62.2%) of the studies included with varying the rate of THA (rang, 10% to 64%) at final follow-up. In 1991, Buckley et al. [50] evaluated the outcomes of 20 hips with ONFH patients in the pre-collapse stages (Marcus I and II) treated with NVBG. After a mean follow-up of 8 years (rang, 2 to 19 years), only two hips (10%) progressed to require a THA. Similarly, Bakx et al. [51] reported the same rate of THA after a mean follow-up of 33 months. In the study by Wang et al. [53]. nine (34%) of 28 hips (ARCO I to III) after allogeneic fibular grafting required THA with a mean follow-up of 25 months. However, in the mean follow-up of 104.5 (rang, 95 to 108) months, they reported 18 (64%) of 28 hips underwent THA at the finally follow-up [39]. Despite THA cannot be avoided in most patients, the time for THA is deferred effectively. Due to the less invasive procedure, the Phemister technique may still be considered as an option for young ONFH patients, but the long-term result needs to be improved.

The trapdoor technique was first reported in 1983 by Meyers et al. [54], and has been used in five of the studies included. After surgical dislocation of the hip, full exposure of the femoral head was established to remove a chondral window from the femoral head, allowing removal of the necrotic bone tissue. Then, the lesion is filled with bone graft, closed, and secured with bioabsorbable pins. Therefore, this technique is more invasive than Phemister technique. The rate of THA after NVBG with trapdoor technique was ranged 4% to 36% in our study. Cheng et al. [26] reported outcomes of this technique in 67 hips with ARCO stage II and III ONFH. After a mean follow-up of 91.2 months, only three (4%) underwent a THA. In the study of Gagala et al. [37], 13 patients had large pre-collapse ARCO IIC and post-collapse ARCO III and IV lesions were treated with autologous osteochondral transfer and morselized bone allografts. At the finally follow-up, Kaplan–Meier survivorship was 61% in this group at three years. The authors therefore concluded that this procedure can be of benefit for patients with pre-collapse or early collapse lesions and largely aims to delay THA in these patients.

In 1994, Rosenwasser et al. [55] described the lightbulb technique, which is similar to the approach of trapdoor technique. After the incision of hip joint capsule, a cortical window is made at the femoral head neck junction. Then, the necrotic bone tissue was completely removed, and the void can be packed by a cortico-cancellous graft or augmented as needed. Our recent study [15] reviewed 64 patients who underwent surgical hip dislocation combined with impacting bone grafts. Patients had between ARCO stage II and III ONFH. The authors reported the conversion rate of THA was 12.86% and concluded that this procedure can be of benefit for patients with retention of the lateral column of the femoral head and hip pain less than one year. Wu et al. [17] reported 50 hips with ARCO stage II and III underwent impaction bone grafting augmented with a wire coil using the lightbulb technique. After a mean follow-up of 109.2 months, 19 hips (38%) had failed and converted to THA at an average of 52.8 months. In summary, this technique, which uses the femoral neck as a conduit for the insertion of bone graft, has shown positive outcomes.

**Fig. 5:**
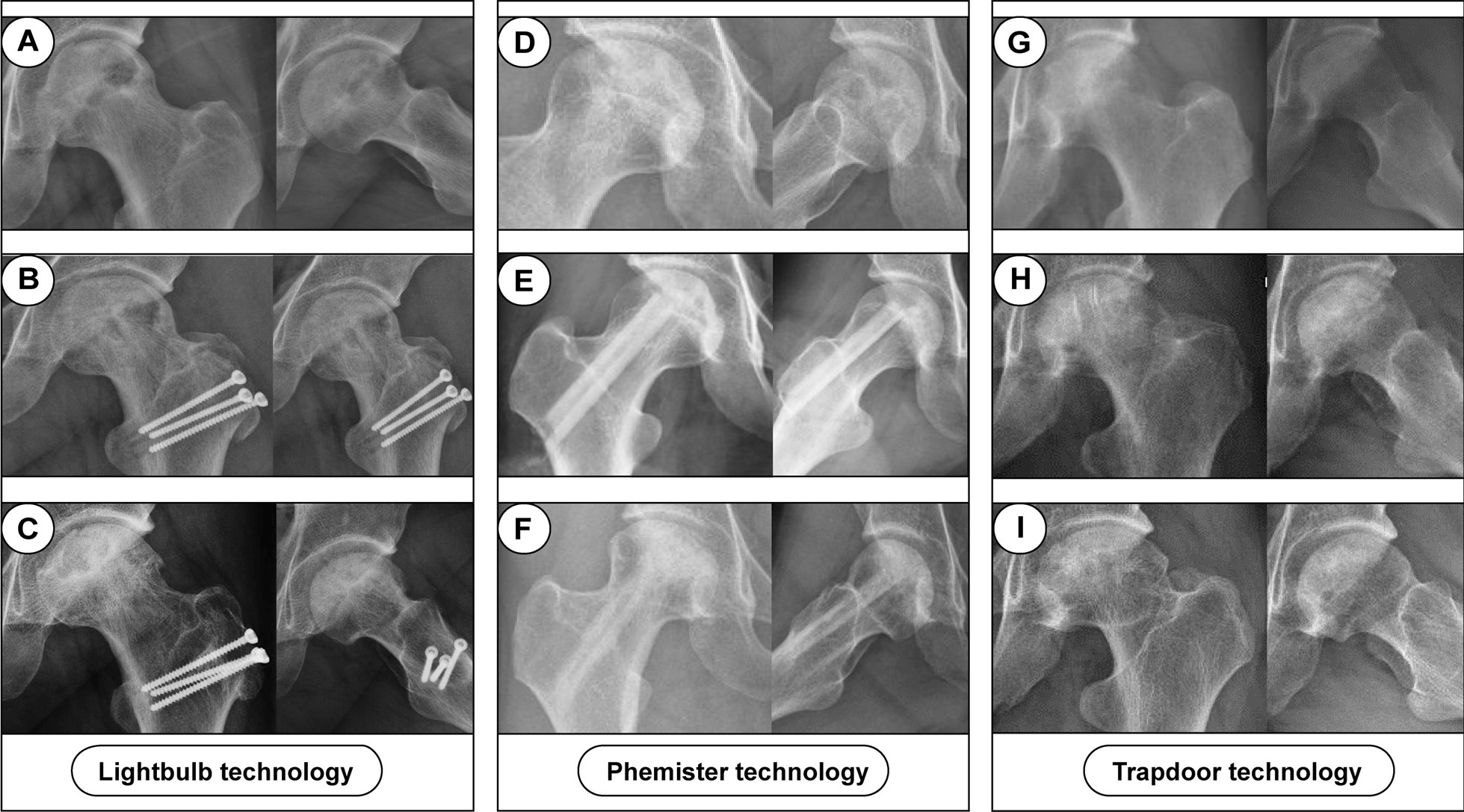
Three patients with ONFH underwent NVBG with three surgery techniques. **A**–**C** A 33-year-old male patient left alcohol-induced ONFH; **B** postoperative radiograph showing that the left femoral head was underwent NVBG with lightbulb technique, and the frog lateral x-ray position was not standard because of pain; **C** radiographs taken 30 months post operation show a restoration of bone. **D**–**F** A 24-year-old female patient right steroid-induced ONFH; **E** postoperative radiograph showing that the right femoral head was underwent NVBG with Phemister technique; **F** radiographs taken 75 months post operation show no further collapse happened; **G**–**I** A 41-year-old male patient left steroid-induced ONFH; **H** postoperative radiograph showing that the left femoral head was underwent NVBG with trapdoor technique; **I** radiographs taken 63 months post operation show the necrotic area was partially repaired

The Phemister, trapdoor and lightbulb techniques provide surgical options for addressing ONFH in pre- and early collapse stages. In the past few decades, these three techniques were tried by our group in the treatment of ONFH with NVBG (Fig. 5). In this study, the rate of conversion to THA with different surgical options was different, and the Phemister group had a higher rate than trapdoor and lightbulb groups. As reported by Keizer et al. [44], the reason for this result may be that the Phemister technique does not provide a good operating view, and thus can’t adequately debride the necrotic bone tissue. In addition, only one study included in this analysis compared the results between trapdoor and lightbulb techniques [26]. In their cohort, 67 patients underwent the trapdoor technique and 72 patients underwent the lightbulb technique. These patients had ARCO stage II and III lesions. At the finally follow-up, three hips (4%) underwent THA in the trapdoor group and nine (12.5%) hips progressed to require a THA in lightbulb group. They concluded that the trapdoor technique was superior compared to the lightbulb technique treatment in patients with ONFH. But owing to the limited number of patients, this trial cannot provide robust support for this conclusion.

Notably, there was no significant difference in the incidence of THA between the ARCO II and ARCO III groups. Over the past several decades, different classification systems, including ARCO system, have been developed to evaluate the stage of severity and prognosis of the disease based on the imaging features of ONFH. However, patients classified with the same severity stage responded differently after similar joint-preserving surgery [40, 42]. The main reason was ARCO system may focus more on the extent of the collapse rather than the regional distribution. As reported by our previous studies [15, 22], in patients with Japanese Osteonecrosis Investigation Committee (JIC) classification type C2, the absence of the lateral column of the femoral head as the main weight-bearing site makes ONFH more likely to progress. However, the information could not be meta-analyzed due to lack of available data from the included studies.

One study compared clinical outcomes and survival rate in the long-term follow-up between non-vascularized autologous fibular graft and an allogeneic fibular graft for the treatment of ONFH [21]. There is no appreciable difference in the rate of conversion to THA between autologous fibular graft group (12%) and allogeneic fibular graft group. What’s more, some studies reported that elderly age tended to lead to worse surgical outcomes [15, 21].

Our study revealed the rate of conversion to a THA after NVBG based on ample published studies, which can help us understand and explore the outcomes in ‘non-expert’ hands. However, there are some limitations of this study. Firstly, the heterogeneity in our study was significant, and the subgroup analysis can’t reduce the heterogeneity. We speculated that the important factors of heterogeneity could be related to different surgeons and surgery procedures, pre-operative stage and extent of necrosis, postoperative rehabilitation, and follow-up time. Secondly, it does also not consider the functional outcomes and purely looks at conversion rate, using conversion to THA as a surrogate for success or failure. Clearly the conversion to THA is a more complex one, with not all failures undergoing conversion. Thirdly, when looking at risk factors for conversions, few papers presented raw data in a way that could be analyzed, detracting from statistical analysis. Therefore, we should be cautious in interpreting the pooled results.

Despite a high degree of heterogeneity among studies, the above studies do clearly indicate that the proper use of NVBG favorable outcomes in patients with early-stage ONFH by deferring the need for THA. Pooled results of 2599 hips and of these nearly 80% with early stage of osteonecrosis, showed that approximately 21% of patients underwent a THA following NVBG at the mean weighted 50.5 months follow-up time. Therefore, NVBG could be an effective hip-preserving alternative for young patients with symptomatic ONFH when patients are appropriately selected, the surgical procedure is accurately performed, and adequate postoperative rehabilitation is provided. The use of various surgical techniques is a matter of surgeon preference and is an area of active investigation. Nevertheless, prospective cohort studies with larger sample sizes are needed in future.

## Supplementary Information

**Additional file 1: Fig. 1**. Proportion Forest plot of studies reporting percentage of hips undergoing THA after NVBG, by follow-up time as analyzed by metaprop.

**Additional file 2: Fig. 2**. Funnel plot of the rate of THA after non-vascularized bone grafts.

**Additional file 3: Table 1**. Quality assessment of cohort studies; **Table 2** Quality assessment of case–control studies.

## Data Availability

The authors declare that all the data supporting the findings of this study are available within the article and its supplementary information files.

## Abbreviations

NVBG: Non-vascularized bone grafting
ONFH: Osteonecrosis of the femoral head
JIC: Japanese Investigation Committee
ARCO: Association Research Circulation Osseous
OR: Odds ratio
