# Supplementary figures and images for: One in five patients require conversion to arthroplasty after non-vascularized bone grafts in patients with osteonecrosis of the femoral head: a systematic review
Source: J Orthop Surg Res. 2023 Jan 31;18:77. doi: 10.1186/s13018-023-03544-8 (PMC9887751; doi:10.1186/s13018-023-03544-8)

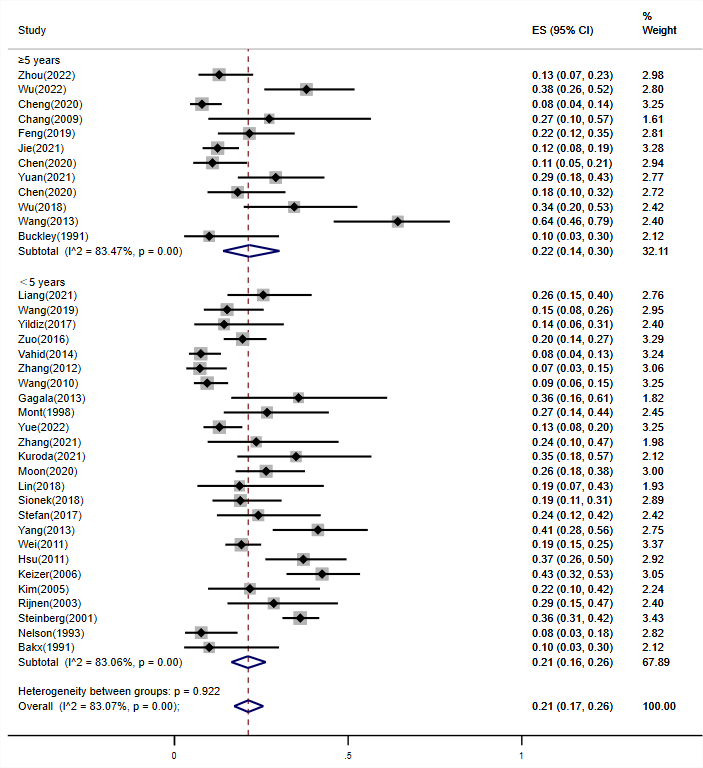

Supplement: Supplementary file 1 — Additional file 1: Fig. 1. Proportion Forest plot of studies reporting percentage of hips undergoing THA after NVBG, by follow-up time as analyzed by metaprop. [file 13018_2023_3544_MOESM1_ESM.tif]

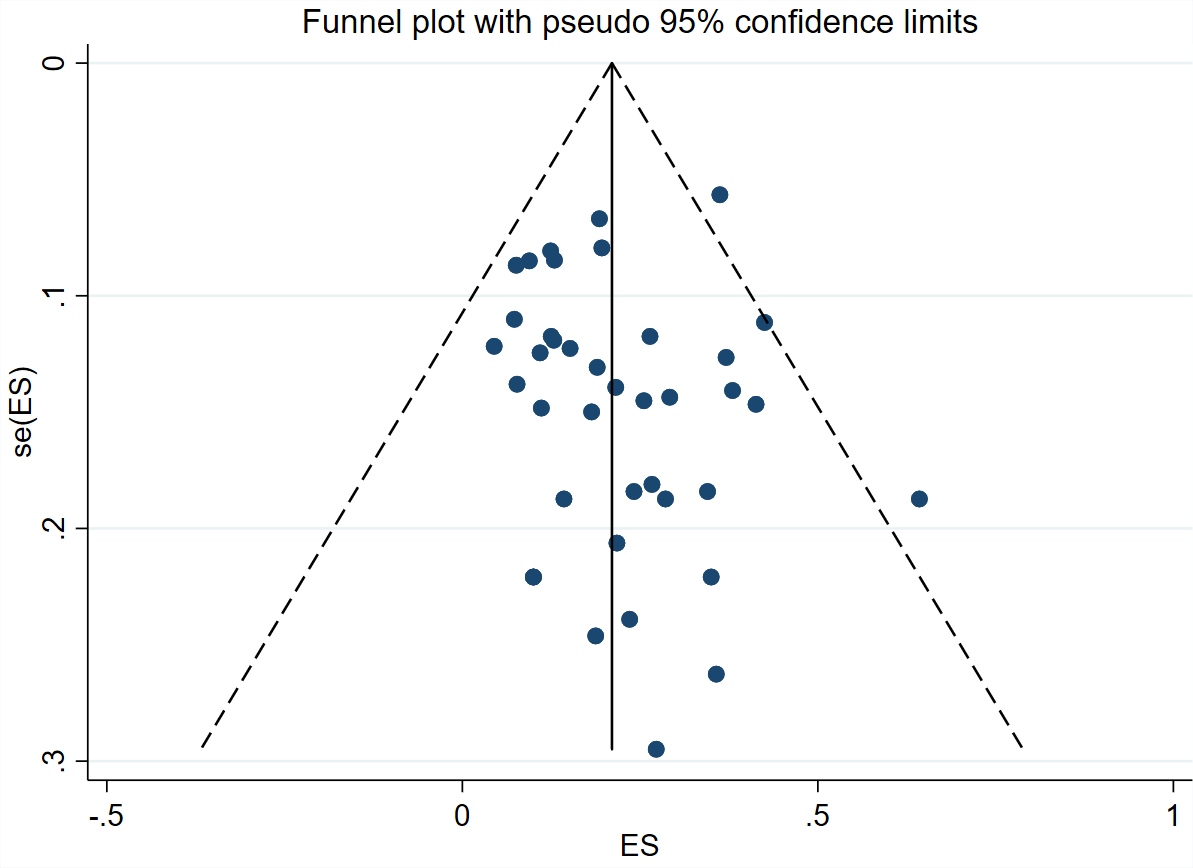

Supplement: Supplementary file 2 — Additional file 2: Fig. 2. Funnel plot of the rate of THA after non-vascularized bone grafts. [file 13018_2023_3544_MOESM2_ESM.tif]
