# Supplementary material for: One in five patients require conversion to arthroplasty after non-vascularized bone grafts in patients with osteonecrosis of the femoral head: a systematic review
Source: J Orthop Surg Res. 2023 Jan 31;18:77. doi: 10.1186/s13018-023-03544-8 (PMC9887751; doi:10.1186/s13018-023-03544-8)
Supplement: Supplementary file 3 — Additional file 3: Table 1. Quality assessment of cohort studies; Table 2 Quality assessment of case–control studies. [file 13018_2023_3544_MOESM3_ESM.pdf]

Supplementary Table 1. Quality assessment of cohort studies.

Study	Level of evidence	Representativeness of the exposed cohort	Selection of the non exposed cohort	Ascertainment of exposure	Demonstration that outcome of interest was not present at start of study	Comparability of cohorts on the basis of the design or analysis	Assessment of outcome	Was follow-up long enough for outcomes to occur	Adequacy of follow-up of cohorts
Zhou2022[10]	4	Yes	NA	Yes	Yes	NA	Yes	Yes	Yes
Yue2022[11]	4	Yes	NA	Yes	Yes	NA	Yes	Yes	Yes
Wu2022[12]	4	Yes	NA	Yes	Yes	NA	Yes	Yes	Yes
Kuroda2021[14]	4	Yes	NA	Yes	Yes	NA	Yes	Yes	Yes
Liang2021[15]	4	Yes	NA	Yes	Yes	NA	Yes	Yes	Yes
Chen2020[17]	4	Yes	NA	Yes	Yes	NA	Yes	Yes	Yes
Yuan2021[18]	4	Yes	NA	Yes	Yes	NA	Yes	Yes	Yes
Chen2020[19]	4	Yes	NA	Yes	Yes	NA	Yes	Yes	Yes
Moon2020[20]	4	Yes	NA	Yes	Yes	NA	Yes	Yes	Yes
Wang2019[23]	4	Yes	NA	Yes	Yes	NA	Yes	Yes	Yes
Wu2018[24]	4	Yes	NA	Yes	Yes	NA	Yes	Yes	Yes
Sionek2018[26]	4	Yes	NA	Yes	Yes	NA	Yes	Yes	Yes
Stefan2017[27]	4	Yes	NA	Yes	Yes	NA	Yes	Yes	Yes
Yildiz2017[28]	4	Yes	NA	Yes	Yes	NA	Yes	Yes	Yes
Zuo2016[29]	4	Yes	NA	Yes	Yes	NA	Yes	Yes	Yes
Vahid2014[30]	4	Yes	NA	Yes	Yes	NA	Yes	Yes	Yes
Zhang2012[33]	4	Yes	NA	Yes	Yes	NA	Yes	Yes	Yes
Wei2011[35]	4	Yes	NA	Yes	Yes	NA	Yes	Yes	Yes
Hsu2011[36]	4	Yes	NA	Yes	Yes	NA	Yes	Yes	Yes
Wang2010[37]	4	Yes	NA	Yes	Yes	NA	Yes	Yes	Yes
Chang2009[38]	4	Yes	NA	Yes	Yes	NA	Yes	Yes	Yes
Keizer2006[39]	4	Yes	NA	Yes	Yes	NA	Yes	Yes	Yes
Rijnen2003[41]	4	Yes	NA	Yes	Yes	NA	Yes	Yes	Yes
Steinberg2001[42]	4	Yes	NA	Yes	Yes	NA	Yes	Yes	Yes
Mont1998[43]	4	Yes	NA	Yes	Yes	NA	Yes	Yes	Yes
Nelson1993[44]	4	Yes	NA	Yes	Yes	NA	Yes	Yes	Yes
Buckley1991[45]	4	Yes	NA	Yes	Yes	NA	Yes	Yes	Yes
Bakx1991[46]	4	Yes	NA	Yes	Yes	NA	Yes	Yes	Yes

Supplementary Table 2. Quality assessment of case-control studies.

Study	Level of evidence	Is the case definition adequate?	Representactiveness of the cases	Selection of controls	Comparability of cases and controls on the basis of the design or analysis	Ascertainment of exposure	Same method of ascertainment for cases and controls	Non-response rate
Jie2021[16]	3a	Yes	Yes	Yes	Yes	Yes	Yes	NA
Zhang2021[13]	3a	Yes	Yes	Yes	Yes	Yes	Yes	Yes
Cheng2020[21]	3a	Yes	Yes	Yes	Yes	Yes	Yes	Yes
Feng2019[22]	3a	Yes	Yes	Yes	Yes	Yes	Yes	Yes
Lin2018[25]	3a	Yes	Yes	Yes	Yes	Yes	Yes	NA
Yang2013[31]	3a	Yes	Yes	Yes	Yes	Yes	Yes	NA
Gagala2013[32]	3a	Yes	Yes	Yes	Yes	Yes	Yes	NA
Wang2013[34]	3a	Yes	Yes	Yes	Yes	Yes	Yes	NA
Kim2005[40]	3a	Yes	Yes	Yes	Yes	Yes	Yes	NA
